# The network of life: genome beginnings and evolution

**DOI:** 10.1098/rstb.2009.0046

**Published:** 2009-08-12

**Authors:** Mark A. Ragan, James O. McInerney, James A. Lake

**Affiliations:** 1 Institute for Molecular Bioscience, and ARC Centre of Excellence in Bioinformatics, The University of Queensland, Brisbane, Australia; 2 Department of Biology, The National University of Ireland, Maynooth, Maynooth Co.Kildare, Ireland; 3 Department of MCD Biology; 4 Department of Human Genetics; 5 Molecular Biology Institute, University of California, Los Angeles, CA, USA

**Keywords:** genome origins, lateral genetic transfer, tree of life, network of life, evolution of viruses, evolution of developmental pathways

The genome sequence is an icon of early twenty-first century biology. Genomes of nearly 2000 cellular organisms, and from many thousands of organelles and viruses, are now in the public domain. For biological research in individual species, the genome sequence increasingly provides the common reference for the application of polymorphism, transcriptomic, proteomic and other genome-scale data to problems of development or disease. More recently, through metagenomic sequencing, the composition and function of ecosystems are being explored at the molecular level. Genomes are now central to genetics, cell and developmental biology, molecular and systems biology, population biology and ecology.

At the same time, one cannot but be struck by the diversity of genomes, both across the living world and, in many cases, within genera or species. Whatever constraints may be imposed by their central role in genetics, functional and cell biology clearly do not stand in the way of genomes ranging in size over eight orders of magnitude and exhibiting remarkable diversity and variation in gene content, gene order and organization. Perhaps most unexpected of all is the substantial decoupling, now known in most, although not all, branches of organismal life, between the phylogenetic histories of individual gene families and what has generally been accepted to be the history of genomes and/or their cellular or organismal host lineages. The tree of life paradigm consolidated by Darwin's *Origin of Species* (1859), but itself arising from a much older tradition of natural history, seems likely to emerge, if at all, from the multi-genome era much more restricted in scope, and subject to many more qualifications, than could have been anticipated a dozen years ago.

The papers in this theme issue of *Philosophical Transactions B* present important perspectives on the origin and evolution of genomes. Key questions include genome beginnings: what preceded genomes? How were the first genomes organized, and according to what principles, by what mechanisms and along what paths did they begin to diversify? Our expert contributors explore genome diversity—among the deepest lineages, and from the highest taxon levels to intriguing individual species—discussing the processes that have shaped, and continue to shape, genomes of bacteria, archaebacteria and eukaryotes. A major theme is lateral (horizontal) genetic transmission, some (perhaps much) of which involves the abundant small elements that pervade the biosphere—viruses, plasmids, gene transfer agents—that can not only shuttle DNA among cellular organisms but also modify it in the process. Do the frequency and impact of lateral transfers on (many) genomes render the tree of life only an approximation—useful in some contexts, less so in others—or indeed is the tree of life paradigm now a barrier to a richer, more integrative understanding of life on Earth?

We have grouped these papers to reflect the chronology and significant evolutionary events in the origin and evolution of genomes. The emerging narrative is fresh with competing concepts and theories. In other words, in the field of genome evolution, the scientific process is alive and healthy.

## 1. Genome beginnings and deep lineages

Life has existed on Earth for much of its 4+ billion year history. Evidence in the form of microscopic fossils, molecular signatures and isotopic abundances arguably indicates that life has inhabited our planet for considerably longer than 3 billion years. New studies point to an increase in oxygen in the atmosphere, a relatively late stage in the evolution of life, starting at least 2.4 billion years ago (reviewed by Falkowski & Isozaki 2008, and by Fischer 2008). Genomic-based rooting studies indicate that the double-membrane prokaryotes, which include the cyanobacteria responsible for Earth's oxygen atmosphere, originated after most of the major prokaryotic groups were established. Thus, cellular life is likely to have been present on Earth considerably earlier.

But genome beginnings extend deeper than this. Before the cenancestor, life must have existed in forms that are difficult, but not impossible, to study using genomic sequences. Many scientists think that the earliest life may have functioned using RNA, rather than proteins, as a catalyst. Progress in this field is rapid. Just recently, ‘self-sustained’ replication of an RNA enzyme was demonstrated (Lincoln & Joyce 2009). It is possible that relics dating from this early world may exist even today in our genomes, in the distinct gene lineages known as informational and operational genes (Rivera *et al*. 1998). These gene classes are found in all eukaryotes and prokaryotes, including humans. Informational genes function in processes such as protein synthesis and RNA transcription, suggesting that they may have originated during an RNA world, whereas the operational genes that perform routine cellular processes such as making cell walls and synthesizing amino acids may have descended from other organisms at different times. Certainly our cells contain genes that are thought to be derived from the RNA world, but our story here starts later, with the last common ancestor of cellular life.

As genomes occur in organisms and every organism contains at least one genome, we begin the story of genome evolution with the *cenancestor*, or last common ancestor of cellular life. Given the extent of lateral gene transfer, the cenancestor was unlikely to have been a single cell, and can better be conceptualized as a population, or populations, containing diverse organisms. Furthermore, these organisms probably did not live at the same time. This may sound incredible, but follows from the way populations evolve. For example, a little more than 15 years ago we learned from Allan Wilson that the mother of all humans lived approximately 200 000 years ago in Africa. This Eve was first identified by comparative sequencing of human mitochondrial genomes, which are maternally inherited. Subsequently, the father of us all was identified by comparative sequencing of human Y chromosomes: he lived only 50 000 years ago, and even if he were Methuselah, he could never have known mitochondrial Eve.

Because different human genes have followed different evolutionary pathways, we must think of the evolution of (multiple) populations. This is even more the case among prokaryotes, whose genes continue to evolve along different, sometimes anastomatizing, paths. If the ‘ancestor of all cellular life’ did not live in any one place or at any one time, there was no single discrete ‘last universal common genome’.

Since the mid-nineteenth century, the living world has been represented as a genealogical tree in which some major lineages extend back in time towards the common cellular ancestor, while others became established only more recently. Lake, Skophammer, Herbold and Servin emphasize the importance of ordering present-day lineages according to time of appearance, and identifying the deepest known lineages—i.e. rooting the tree of life. In this way, it ‘becomes possible to relate genetic, biochemical, ultrastructural and behavioural innovations to geological, paleontological and climatological events, thereby allowing one to trace the interdependent histories of the Earth and its microbiota, and to test theories for the order of appearance of novel biological innovations’—for example, the order of appearance of methanogenesis, respiration and substrate-level phosphorylation, heterotrophy versus autotrophy, single versus double-delimiting membranes and thermophily versus mesophily. These advantages remain even if—as other papers in this theme issue discuss in detail—genomic or cellular relationships turn out to be better represented as a ring, network or other topology.

Lake and colleagues use insertion–deletion events (indels) in eight sets of paralogous genes to exclude the root from many regions of the tree (or network) of life as presently known. In this way, they localize the root to the base of two major lineages, one consisting of the actinobacteria and the double-membrane (Gram-negative) prokaryotes, the other consisting of archaebacteria and firmicutes. Their approach is progressive, in that the root (or root area) can be excluded from additional regions as new genomic data appear (i.e. as we realize that certain regions are not the most ancient) without having to re-analyse everything that has gone before. Using this approach, it can already be concluded that double-membrane prokaryotes (including the cyanobacteria, mentioned above) were derived from simpler single-membrane prokaryotes, and that members of the cenancestral population were enclosed by ester-linked lipid membranes and surrounded by a peptidoglycan layer. Components of the toolkit for archaebacterial lipid biosynthesis seem also to have been present in the common ancestral population, or at least immediately ancestral to the bacilli and archaebacteria.

## 2. Lateral genetic transfer

Descent with modification has long been accepted as the framework within which the transmission of genetic determinants is best explained, at least in morphologically complex eukaryotes. As for bacteria and archaebacteria, the genomic era has thrown up data that do not fit a straightforward vertical-descent model. A surprising number of gene trees are, in part, topologically discordant with each other and/or with accepted organismal relationships. Many genes are distributed across genomes and taxa in patterns that cannot be reconciled parsimoniously with a purely vertical pattern of genetic transmission and gene loss, and sometimes exhibit compositional features (e.g. dinucleotide content) distinct from the surrounding genome and characteristic of more-distant taxa. Further, there is unequivocal evidence (e.g. from the spread of antibiotic resistance) that genetic information is readily transmitted laterally within some populations, particularly but not exclusively in strongly selective environments; some processes are well characterized at the molecular level, and a plenitude of potential vectors (the *mobilome*: Frost *et al*. 2005) appears to be available.

Although there is considerable consensus that these lines of evidence point to lateral genetic transfer (LGT) as potentially widespread and physiologically important, many issues remain unresolved. Ragan and Beiko organize these by *Process & mechanism*, *Quantification*, and *Impact*. It is not known, for example, how much each process (transformation, transduction, conjugation) or each type of vector contributes to LGT, either globally or within a species; vectors can leave telltale clues, but in some cases interpreting these clues has proved to be trickier than expected. Structure is apparent in the living world, implying the existence of constraints to exchange: are these mainly external to organisms (lack of contact, lack of genes to work with, selective pressure for small genome sizes), or mainly internal (barriers to uptake and recombination, complexity of integrating new genes into the host network)? Lateral origins have been claimed for genomic regions of diverse size, from a few nucleotides to complete chromosomes; but interestingly, *domons* (exonic regions corresponding to protein domains, hence presumably to component units of function) do not appear to be a primary unit of LGT (Chan *et al*. 2009). Many genes have mixed heritage, and these authors urge greater precision in describing them as *vertical* or *lateral*, *concordant* or *discordant*.

Ragan and Beiko ask how we should think about and express the extent of LGT: as the proportion of genomes affected by LGT (close to 100%), the proportion of genes with at least one lateral event in their history (estimates in the range 25–50% or more are not infrequent) or the proportion of internal graph edges that are concordant with a reference tree (perhaps 5–15% for subsets of orthologous gene trees). More generally, is it better to count transfers, or to model transfer? Are different evidence types—say, gene trees and distribution data—complementary? How much LGT is undetectable? Can the biological sources of transfers be identified? How long do laterally transferred sequences persist in a genome? Can ancient metabolisms be reconstructed? If LGT has been frequent, then physiology (hence ecological niche) has presumably changed repeatedly over time.

Introgressed genetic material must be connected into the cellular networks of genetic regulation, molecular interactions, metabolite flow and energetics. Although the field is in its infancy, Ragan and Beiko summarize the ‘network view’ of cell as: (i) cellular networks contain both highly and weakly connected nodes; (ii) a species-specific *core* subset of nodes is present in all strains of a species, while other nodes may be found in some or few strains; (iii) core nodes are chromosomal, whereas peripheral nodes may or may not be; (iv) core nodes typically describe functional units, e.g. operons or macromolecular complexes; (v) core nodes tend to be more highly connected and more highly expressed than peripheral nodes; (vi) core-node genes accumulate point mutations more slowly than do peripheral-node genes; (vii) peripheral nodes are more often implicated in functions that are directly affected by the environment; and (viii) networks evolve by the addition of peripheral nodes. It remains to be seen how general these prove to be: do all prokaryotes have a more-or-less stable, taxon-specific core genome? What factors determine the size and composition of the peripheral genome, and how large is the pan-genome? The latter questions become important in the arguments of Dagan and Martin, below.

At least in bacteria and archaea, LGT is often portrayed solely as a confounding factor for genome phylogeny: it disrupts the ‘workable alignment of phenetics and cladistic practices’ (Doolittle, see below). A recurring theme within this theme issue is that this, by itself, is too narrow a view. LGT is a central modality of genome evolution, and treating it purely as a distraction from vertical (parent-to-offspring) transmission hinders us from appreciating the broader fabric of evolution, specifically the plurality of mechanism and pattern beyond a unitary tree of life. Fournier, Huang and Gogarten argue further that LGT events, once properly recognized against the background ‘plurality signal’, can actually benefit phylogenetic reconstruction, specifically by allowing dates (relative or absolute) to be assigned to speciation events. Sudden radiations are unexpected under a steady-state model of extinction and speciation, and their presence therefore requires explanation. For example, the radiation of major lineages deep in the bacterial tree may have resulted from niche expansion following a mass extinction some 3.8–4.1 Gyr ago.

Transporters originating from chlamydial endosymbionts may have contributed to a favourable environment for the establishment of plastids via cyanobacterial endosymbiosis; more broadly, novel functions are sometimes seen to have been recruited from multiple sources in multiple events (‘concerted gene recruitment’: Huang & Gogarten 2008). Two enzymes that activate acetate for input into acetoclastic methanogenesis in *Methanosarcina* appear to have originated in cellulolytic clostridia; directionality of the transfer is clear from the phyletic distribution, and from the adjacency of their genes in both source and recipient genomes. Moreover, modern representatives of the two taxa commonly co-occur in aquatic environments. Fournier *et al*. therefore infer that *Methanosarcina* began to contribute to acetoclastic methane production in the environment only after cellulose (e.g. aquatic plants) became abundant in aquatic environments, i.e. later than 475 Myr ago.

## 3. The tree or network of life

Darwin's hypothesis—that all modern organisms are historically related via genetic descent, with modification, from one or a very small number of common ancestors—has had the profoundest intellectual, scientific and social consequences over the last 150 years. One of these has been the widespread acceptance—variously as metaphor, hypothesis, model or true historical description—of a single unitary tree of life. The growing appreciation that the tree of life may oversimplify reality, or indeed be fundamentally incorrect, necessarily bears profound implications across the hard sciences, and well beyond.

Haggerty, Martin, Fitzpatrick and McInerney illustrate the extent of disjunction possible between gene and genome histories. Working with 27 complete genomes from the well-studied YESS (*Yersinia*, *Escherichia*, *Salmonella*, *Shigella*) group of enteric bacteria, the authors probe different subsets of the data: the entire complement of 16S rRNA genes, three commonly analysed housekeeping genes and their concatenation, a concatenated nucleotide alignment of all 1408 unambiguously orthologous genes, and a supertree of the same 1408 genes. Within the YESS group they find three major 16S rRNA subtrees corresponding to a *Yersinia* type, a *Salmonella* type and an *Escherichia*/*Shigella* type. Similar genus-level subtrees were recovered from the other approaches; but beyond this, agreement largely breaks down. Within-species relationships are inconsistent among trees inferred from the three individual protein-coding genes; between each of these and the tree inferred from their concatenated sequences; and with the rRNA and 1408-gene trees. The two 1408-gene trees (and minimum-evolution tree of their concatenated nucleotide data, and the supertree) are topologically identical within *Salmonella* and disagree only slightly within the *Escherichia*/*Shigella* clade, but conflict seriously within *Yersinia*. Compared with other taxa the YESS genomes are well sampled, and there is no reason to believe that their genes are particularly refractory to phylogenetic analysis; yet within each of these three genus-level groups, different standard samples of the YESS genomes fail to converge on a single consistent signal, presumably due to frequent LGT. Thus, the concept *genome tree* does not appear to be helpful, or even especially meaningful, in this important taxon.

Dagan and Martin lead us farther into the network paradigm of genome evolution. As introduced above, genomes of bacteria and archaea often consist of a taxon-specific set of core genes, and a potentially much larger set of peripheral genes associated more transiently with, and shared among, genomes. For any genome, the size of its peripheral gene set can vary over time but is constrained by physical factors and the necessity of maintaining transcriptional control (Gagen & Mattick 2005), and except in specialized cases of genome degeneracy (e.g. intracellular parasites: Moran 2003) there is little reason to think that ancestral genomes were, on average, significantly larger or smaller than present-day examples. Any accurately inferred gene tree can be reconciled with a discordant external expectation (reference phylogeny) by assuming some number of ancient gene duplications with subsequent differential loss. But each time we assume this, we assume that an additional gene was present in the genome of that ancestor (Dagan & Martin 2007). Over all such cases, this would lead to unrealistically large ancestral genome sizes—the ‘genome of Eden’ of Doolittle *et al*. (2003). By contrast, assuming LGT reduces the inferred sizes of ancestral genomes.

To capture this broader picture of genome dynamics (including gene loss and LGT), we need to describe and depict phylogenetic relationships of all genes, not only the (often relatively small) taxon-specific conserved genomic core; and for this we require a network perspective and tools. Dagan and Martin depict a network of vertical inheritance and lateral exchange for 181 prokaryotic genomes, as well as sub-networks within and among internal and external nodes, but allow that it ‘will probably take some time before LGT among prokaryotes and the endosymbiotic origins of chloroplasts and mitochondria can be reconstructed at the computer in a unified framework that starts with genome sequences and ends up with a network that is both readily printable and readily interpretable’.

These authors extend this perspective to eukaryotes, where the archaebacterial nature of the genetic apparatus does not predict the eubacterial nature of energy metabolism. LGT is known but is much less extensive than among prokaryotes: even in *Entamoeba*, which harbours prokaryotic endosymbionts, only 1–2% of nuclear genes might have been acquired laterally (Loftus *et al*. 2005). On the other hand, large-scale gene transfer has occurred from endosymbionts to host nucleus during the establishment of mitochondria and plastids, and many more eukaryotic nuclear genes share homologs with bacteria than with archaebacteria. Which among these came from the proto-organelles? The authors argue that unlike the few ‘oddly branching copies of highly similar genes’ currently thought to have been transferred more recently among eukaryotes, genes from the proto-organellar endosymbionts contributed fundamentally new physiology, e.g. mitochondrial ATP synthesis and photosynthesis. In this Dagan and Martin support the proposal of Allen (2003) that the retention of genomes in organelles has allowed redox-dependent regulation of gene expression.

Doolittle situates the tree of life in broader scientific, philosophical and social contexts. He argues (following Panchen 1992 and Doolittle & Bapteste 2007) that Darwin viewed the tree as a hypothesis about evolutionary process and the consequent patterns among organismal form and function. Darwin mobilized data on animal breeding, comparative anatomy and especially population dynamics, concluding from these that selection caused descent to be with modification and speciation, *ergo* tree-like. The tree has proved to be an adequate representation of genealogical history within morphologically complex eukaryotes in particular—the main focus of phylogenetics (and later, molecular phylogenetics) during their formative years. Of course neither Darwin nor his contemporaries (before 1859, at least) knew much about microbes or microbial biodiversity. But as knowledge grew, so did scepticism that, in view of their morphological simplicity and physiological flexibility, prokaryotes could ever be brought into a phylogenetic framework (Woese 1987). In the end, the ‘heroic effort’ of Woese and colleagues to extend the tree of life to bacteria and archaea was defeated because these organisms have not, in reality, evolved (purely) by descent with modification on a unitary tree.

Might there nonetheless be a unitary, hierarchical, well-behaved tree of cells? Doolittle characterizes this as a watering-down of Darwin's theory, and an evasion of the true aim of phylogenetics, i.e. the reconstruction of evolutionary paths. Just as not everything in evolution can be explained by natural selection (Gould's *process pluralism*), similarly not everything in phylogeny boils down to trees (Doolittle's *pattern pluralism*). The author proposes, as a general formulation, that ‘genetic mechanisms (broadly construed) and population and ecological process (broadly construed) that we already for the most part understand, operating over enormous time, are responsible for the diversity of life we see around us, and for the adaptedness of living things’. We no longer have a universal hierarchical classification or a unitary, bifurcating tree of life, but our toolkit still contains powerful methodologies (genetics, population biology, ecological theory) to explain the history of life and the diversity of the natural world.

## 4. Genomic origins of eukaryotes

Eukaryotes are unique in possessing a membrane-delimited compartment, the nucleus, in which the chromosomes are found. Eukaryotes may further contain additional types of membrane-delimited, genome-containing compartment, e.g. mitochondria and plastids. Our authors argue that mitochondria are ancestral in the eukaryotic lineage and were subsequently lost or modified in various lineages, although alternative scenarios have been proposed. There is near-unanimity that these two organelles, at least, have descended from free-living bacteria that, analogously with present-day examples, became entrapped in an endosymbiotic relationship. However, modern free-living relatives of the likely proto-organelles have genomes 10- to 100-fold larger than most mitochondria and plastids. Were the missing genes simply lost, or were they transferred to the nuclear genome (which, to be sure, is extensively ‘prokaryotic’ as assessed by sequence similarity and domain content); and why were specific genes, or indeed any genes at all, retained in what is now the organellar nucleus? Have other non-eukaryotic lineages been merged into the eukaryotic nucleus without leaving a telltale organellar vestige? Several papers in the earlier sections discuss early origins of major extant lineages, including that of the eukaryotic nucleus. Here, two papers explicitly discuss the origin of eukaryotes and their phylogenetic relationship to prokaryotes, and their complex morphology.

Foster, Cox and Embley examine relationships among the most ancient lineages; however, unlike previous authors they focus on the origin of the eukaryotic nuclear lineage. Two hypotheses in particular have received wide attention: the three-domain tree, in which eukaryotes are usually presented as the sister lineage to archaea, and the eocyte tree, in which eukaryotes arise from within archaea as the sister lineage to crenarchaeotes. The application of molecular phylogenetic approaches to such ancient events is intrinsically difficult: signal is not only weak, but potentially confounded by the inability of evolutionary models and phylogenetic methods to correct for site saturation, across-site and across-tree rate variation, compositional heterogeneity and unrecognized homoplasy. Using computational simulations, the authors find that substitutional saturation can be delayed by accounting for among-site rate variation, potentially leaving signal in the data. They develop a model that attempts to accommodate among-site and across-tree compositional heterogeneity, and apply it in conjunction with a number of sophisticated inference methods.

With an rRNA-sequence dataset, diverse methods support the three-domain tree in the absence of correction for compositional heterogeneity, but support increases for the eocyte tree using their corrective model. A concatenated 41-protein amino acid dataset that emphasizes core genetic machinery (slightly expanded from Cox *et al*. 2008), when recoded in order to minimize homoplasy in the analysis of deep-level phylogenetic relationships, supports the eocyte tree with or without their model. Four groups (bacteria, euryarchaeotes, crenarchaeotes, eukaryotes) are individually recovered as monophyletic in most analyses, although interestingly, using standard amino acid coding, a method that is considered to be relatively resistant to long-branch attraction artefacts, reconstructs euryarchaeota as a paraphyletic group, with *Pyrococcus* as the sister group of crenarchaeota and eukaryotes. If real, this implies that crenarchaeotes (i.e. eocytes) plus eukaryotes arose from within the euryarchaea.

Complex morphology and the complex developmental pathways that bring it about are the other eukaryotic characteristics discussed in this theme issue. Erwin focuses on the origin of the bilaterian genomic toolkit—the transcription factors, signalling-pathway genes and other regulatory elements once thought to be characteristic of bilaterally symmetric animals, and associated with their diverse cell types and complex morphologies. Many of these genes have now been found in genomes of non-bilaterian animals including choanoflagellates, placozoan, cnidarian and sponges, more-ancient clades that lack diverse cell types and complex morphogenesis. What was the ancestral role of these genes, and how were they assembled into a functional developmental system in bilaterians?

Early bilaterians (predating the divergence of protostomes from deuterostomes) may not have been particularly complex, with cell-type specification and regional patterning but not complex morphogenetic development (Erwin & Davidson 2002). Modern bilaterians have six major signalling pathways (*Wnt*, TGF-β, Notch, Hedgehog, Jak/STAT and RT), all of which, together with a diversity of transcription factors, are present in cnidarians as well. Thus, the last common ancestor of metazoa could ‘specify multiple cell types, establish body axes, array different cell types along these axes and produce multicellular structures’ although it seems to have lacked the ‘regulatory complexity and depth of transcription factors and microRNAs required to produce complex gene regulatory networks’ and hence complex morphologies. In this context Erwin reminds us of the Eidacarian fauna, most of which is believed to have predated the protostome–deuterostome divergence; we might think of their diverse and remarkable morphologies as outcomes of a critical period in the evolution of regulatory networks, as more and different types of regulatory components were recruited into networks and different degrees and patterns of connectivity were explored.

Even deeper in the animal tree, genomic sequencing of the choanoflagellate *Monosiga brevicollis* has uncovered a considerable diversity of cell adhesion, extracellular matrix, signal transduction and cell differentiation elements including 78 protein domains shared exclusively by choanoflagellates and metazoa (albeit often in different protein contexts, hence presumably without the same functionality, e.g. in cell–cell adhesion). The presence of many ‘bilaterian’ developmental tools in the morphological toolkits available to earlier, simpler animals reinforces the view that the original role of these genes and regulatory networks was in the formation of specialized cell types in specific body regions, not necessarily in producing complex multicellular structures (Erwin & Davidson 2002). Developmental control of pattern formation was then later intercalated into these simpler networks.

## 5. Genome evolution at the community level … and beyond

With the role of LGT in prokaryotic evolution now more fully appreciated, a new field of microbiology is emerging in which the traditionally recognized mechanisms of genetic transfer—conjugation, transformation and bacteriophage transduction—are viewed from a perspective strongly grounded in population biology, ecology and genomics. The final two papers in this theme issue consider the evolutionary competitions between plasmids and the genomes of their hosts. These interactions—some of them quite unanticipated—are opening a new window into the community evolution of genomes.

Norman, Hansen and Sørensen address questions involving the metabolic and genetic interactions in prokaryotes, and their effects on the evolution of these organisms. Unlike the situation in eukaryotes, where cell division and genetic exchange can be coupled, gene exchange in prokaryotes is not generally coupled with cell division. Consequently, additional mechanisms of LGT (or simply gene transfer) are necessary. Prokaryotes frequently occupy microenvironmental niches and live in complex communities where interaction is frequent and genetic transfer can be common. Not surprisingly, genetic transfer has been shown to occur most frequently between organisms that share the same environment (Jain *et al*. 2003), and genes are found in mobile genetic elements transferred among members of these communities, besides being transferred more directly. Norman and colleagues distinguish between genes present in the mobile genetic elements that form the ‘communal gene pool’, and genes distinctive of specific prokaryotic organisms that form ‘private gene pools’.

These authors focus on barriers to gene transfer within the communal gene pool. They emphasize mechanistic, physical, biofilm-related and selective barriers to gene transfer, and provide an intriguing analysis of the roles of mobile genetic elements. Particularly interesting are conjugative plasmids (Helinski 2004), extra-chromosomal circular, double-stranded DNAs that can replicate autonomously and are thus semi-independent of the host chromosome. The authors’ description of selective pressures that have driven the evolutionary wars between conjugative plasmids and their hosts broadens our appreciation of the significance of copy number, conjugation machinery and the modularity of plasmid genetic organization.

We have already encountered viruses as part of the mobilome, i.e. as mobile elements that can shuttle genetic material within and between related sets of organisms. Brüssow encourages us to see viruses as an extension of the bacterial pan-genome, a gigantic ‘repository for storing and sharing genes among their microbial hosts’, and probably ‘a form of infectious sex for bacteria’. And if an extension of the bacterial pan-genome, are viruses part of the living world? Rejecting extreme views of viruses as ‘complex crystals’ or as hyper-reduced, parasitic bacterial genomes, the author posits that ‘in view of the polythetic nature of current life definitions, viruses cannot be dismissed as non-living material’. If viruses fall outside our definition of life, perhaps it is only because our conceptualization of life (and indeed of viruses) is too restrictive. Biology has a ‘fuzzy border’ and phages are hardly as extreme as, say, viroids or prions.

So should we welcome viruses into the tree (or network) of life? The case is perhaps strongest for the amoeba-associated mimivirus (Raoult & Forterre 2008), which has a 1.2-Mbp genome, encodes some 1262 putative genes and six tRNAs, stains Gram-positive and has its own satellite virus. Phylogenetic analysis of a concatenated mimivirus dataset (sequences of seven proteins, six of which are involved in information processing) positions these proteins basally in the eukaryotic nuclear lineage. More generally, though, Brüssow argues that in some respects phages ‘follow their own evolutionary trajectories’ in which there are ‘substantial elements of vertical evolution’ (Brüssow & Desiere 2001); although we cannot speak of a (meaningful) unitary tree of viruses, it is possible to discern among them gradients of sequence similarity; protein sequence identity extending even to RNA viruses; instances of conserved gene order; and shared protein folds, most notably the capsid fold. Some viral proteins (if not viruses themselves) appear to have predated the common cellular ancestor, and Forterre (1999, 2006) has proposed that not only key genes specifying DNA replication, but the double-stranded DNA genomes themselves, arose separately in three RNA-based cells (the proto-bacteria, archaebacteria and eukaryotes) by acquisition of three distinct DNA viruses.

## 6. Envoi

For some 150 years, Darwin's *Origin of Species* has been a cornerstone of the modern intellectual framework within which questions about the origin and evolution of organisms have been formulated. These questions themselves have evolved—some have been answered, others reformulated or rendered irrelevant, while new ones have arisen—as the framework has been enriched by the appearance and development of new disciplines (microbiology, genetics, biochemistry) and technologies (electron microscopy, DNA sequencing, scientific computing). The rapid growth of genome-sequence data since the mid-1990s is now providing unprecedented detail on the genetic basis of life, and not surprisingly is catalysing the most fundamental re-evaluation of origins and evolution since Darwin's day. Several papers in this theme issue argue that Darwin's tree of life is now best seen as an approximation—one quite adequate as a description of some parts of the living world (e.g. morphologically complex eukaryotes), but less helpful elsewhere (e.g. viruses and many prokaryotes); indeed, one of our authors goes farther, proclaiming the ‘demise’ of Darwin's tree as a hypothesis on the diversity and seeming naturalness of hierarchical arrangements of groups of living organisms. It is now more complicated to ask—as do two papers in this issue—the location of the root of the tree of life, and whether there exist three, or four, major groups of organisms on this planet. Genomes are more dynamic than had been assumed, lineages less coherent through time, evolution less tree-like.

Science remains an ongoing process, and further application of existing technologies (not to mention those now in development, and others currently unanticipated) will generate new data highly relevant to questions of origins and evolution, for example, on genome dynamics within populations, the flow of genetic material through viruses and virus-like particles, and the diversity of organismal life—including deep taxa—currently not amenable to laboratory culture, hence unknown. Not all current knowledge—of the frequency of LGT, for example, or the evolution of molecular-interaction networks—is likely to generalize as sampling becomes less biased towards laboratory, pathogenic or economically interesting strains.

The 11 papers that follow in this theme issue, The Network of Life: Genome Beginnings and Evolution, offer diverse perspectives and arguments, frequently complementary, occasionally conflicting, always thought-provoking. We commend them to you as a splendid example of the scientific process at work.
